# The relationship between autoimmune diseases and temporomandibular disorders: A study combined with the GEO database

**DOI:** 10.1097/MD.0000000000046760

**Published:** 2025-12-26

**Authors:** Feihan Gu, Weimin Bao, Mingyu Zhao, Wenkai Huang, Erli Wu, Xiaorong Yan, Hu Zheng, Yuanyin Wang, Ran Chen

**Affiliations:** a College and Hospital of Stomatology, Anhui Medical University, Key Lab. of Oral Diseases Research of Anhui Province, Hefei, China.

**Keywords:** autoimmune diseases, genome-wide association study, Mendelian randomization, single-nucleotide polymorphisms, temporomandibular disorders

## Abstract

The aim of this study was to investigate the causal relationship between various autoimmune diseases (AIDs) and temporomandibular joint disorders (TMD) using Mendelian randomized (MR) analysis. Using publicly accessible statistics from genome-wide association studies (GWAS) statistics, we conducted a 2-sample MR analysis to examine the relationship between 5 prevalent AIDs and TMD, including rheumatoid arthritis (RA), multiple sclerosis (MS), ankylosing spondylitis (AS), and systemic lupus erythematosus (SLE) and psoriasis. The inverse variance weighted (IVW) was chosen as the main analysis method. The impacts of heterogeneity and pleiotropy were evaluated to validate the results. Finally, we verify the results using the bioinformatics method. Our MR analysis found RA (inverse variance weighted odds ratio (OR) = 1.087, 95% confidence interval (CI) = 1.027–1.151, *P* = .004) and MS (OR = 1.081; 95% CI: 1.029–1.135, *P* = .002) and AS (OR = 1.355; 95% CI: 1.037–1.772, *P* = .026) may increase the risk of TMD. Additionally, a reverse causal connection was found between TMD and RA (OR = 1.099; 95% CI: 1.006–1.200, *P* = .027). In addition, bioinformatics analysis revealed common DEGs between RA and TMD, highlighting shared molecular mechanisms that may underlie their association. The results of this study offer compelling evidence that some AIDs, including RA, MS, and AS, increase the likelihood of developing TMD. These results offer fresh perspectives on the fundamental etiological mechanisms of TMD and function as a guide for future clinical interventions aimed at AIDs patients, including early screening and TMD prevention.

## 1. Introduction

The term “temporomandibular disorder” (TMD) describes a collection of illnesses that affect the masticatory muscles, the temporomandibular joint (TMJ), and other musculoskeletal structures in the head and neck.^[1]^ According to reports, roughly 10% of adults over the age of 18 have TMD. It mainly occurs in young and middle-aged adults, and is about twice as frequent in women as in men.^[2]^ After back pain, TMD is the second most prevalent chronic pain disease in the general population. It includes a complex set of conditions, manifesting with limitations in mouth opening and jaw pain, influencing chewing, and facial expression, eating, speaking.^[3]^ Research suggests that TMJ dysfunction may be associated not only with mechanical abnormalities, trauma, and infections but also with underlying autoimmune diseases (AIDs) such as rheumatoid arthritis (RA), psoriatic arthritis, and ankylosing spondylitis (AS).^[4]^ Therefore, regular TMD screening should be performed on patients with known AIDs. Since there is no treatment that can reverse chronic TMJ injuries once they are established, early diagnosis and prevention are critical.

Autoimmune diseases (AIDs) are a widespread disorder characterized by a disorder of the immune system that causes B and T cells to respond abnormally to normal components of the host.^[5]^ Common AIDs include RA, multiple sclerosis (MS), AS, systemic lupus erythematosus (SLE), and psoriasis. The existence of comorbidity between TMD and other AIDs has been documented in an increasing number of observational studies in recent years.^[6]^ For example, numerous research have demonstrated that there may be a correlation between RA and TMD, as well as a risk factor relationship between AS and TMD.^[7,8]^ A meta-analysis performed in 2022 revealed a relationship between MS and TMD and suggested that MS could be a risk factor for TMD.^[9]^ Given that the frequency of both conditions is rising annually, it is critical to ascertain whether there is a causal connection between various AIDs and TMD. Prior research has primarily been observational, making it vulnerable to bias in selection, residual confounding, and reverse causation. Additionally, randomized controlled trials (RCTs) have difficulties with regard to sample size and long-term follow-up. Therefore, developing new epidemiological methods is critically needed.

As genomics and genetic epidemiology have advanced, numerous genetic variations linked to human diseases have been found. Recently, genetic variants have been used as instrumental variables (IVs) in Mendelian randomization (MR), which is a prominent technique for determining plausible causal links between exposures and outcomes. Finding IVs that can function as a connecting factor using the 3 core presumptions of IVs – association, independence, and exclusivity – is the core of MR analysis.^[10]^ MR investigations take advantage of the random distribution of genetic variation, the stability of allele frequencies in the face of illness, and the lack of mutual influence between various traits. This method is not constrained by the shortcomings of RCTs and observational studies.

In the current research, we used MR analysis to investigate possible causal links between 5 prevalent AIDs and TMD. Additionally, our goal was to elucidate their interaction and provide fresh perspectives on the underlying mechanisms.

## 2. Materials and methods

### 2.1. Study design

**Figure 1.**
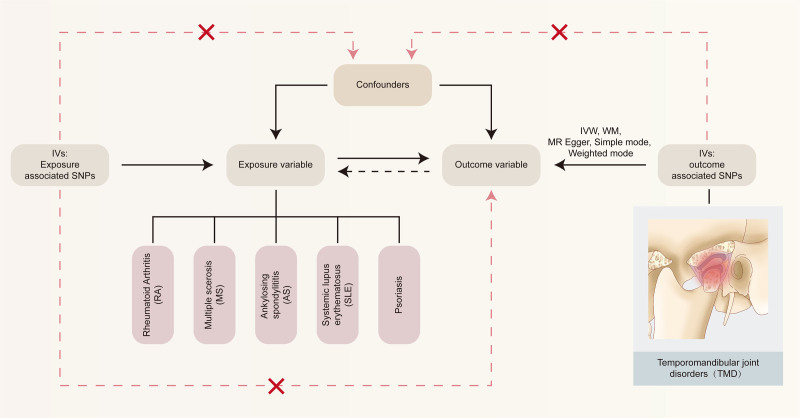
Overall design and flow chart of the present study. AS = ankylosing spondylitis, IV = instrumental variable, IVW = inverse variance weighted, MR = Mendelian randomization, MS = multiple sclerosis, RA = rheumatoid arthritis, SLE = systemic lupus erythematosus, SNP = single-nucleotide polymorphism, TMD = temporomandibular joint disorders, WM = weighted median.

For a 2-sample MR study, multiple single-nucleotide polymorphisms (SNPs) suggesting genetic variation were selected as IVs. Three of the following assumptions were chosen: IVs are strongly and directly associated with exposure; IVs are not affected by any confounding variables; and the impact of IVs on outcomes is solely dependent on exposure.^[11]^ Figure 1 depicted the comprehensive verification procedure for these theories.

### 2.2. Data source

This study largely derived its summary statistics for the genome-wide association studies (GWAS) on AIDs from 2 reliable databases: the FinnGen Consortium (http://www.finngen.fi) and the IEU Open GWAS Project (https://gwas.mrcieu.ac.uk/). Particularly, information for the exposure group contained RA (14,361 cases and 43,923 controls), MS (4888 cases and 10395controls), AS (9069 cases and 1550 controls), SLE (5201 cases and 9066 controls), psoriasis (5314 cases and 4,57,619 controls). Data for TMD was extracted from the FinnGen database, which included 2730 clinically diagnosed cases and 1,31,550 controls. All of the study participants were of European descent. Finally, because autoimmune diseases and TMD came from different consortia, there was no sample overlap. The characteristics of the GWAS consortiums used for each variable are presented in Table 1.

**Table 1:** Details of the studies in the Mendelian randomization analysis.

Phenotype	Consortium	Ethnicity	Sample size	Year	Web source	PMID
TMD	FinnGen	European	1,34,280	2021	https://gwas.mrcieu.ac.uk/datasets/finn-b-TEMPOROMANDIB/	–
RA	EBI	European	58,284	2013	https://gwas.mrcieu.ac.uk/datasets/ebi-a-GCST002318/	24390342
MS	EBI	European	15,283	2016	https://gwas.mrcieu.ac.uk/datasets/ebi-a-GCST003566/	27386562
AS	EBI	European	22,647	2013	https://gwas.mrcieu.ac.uk/datasets/ebi-a-GCST005529/	23749187
SLE	EBI	European	14,267	2015	https://gwas.mrcieu.ac.uk/datasets/ebi-a-GCST003156/	26502338
Psoriasis	MRC-IEU	European	4,62,933	2018	https://gwas.mrcieu.ac.uk/datasets/ukb-b-10537/	–

AS = ankylosing spondylitis, EBI = European Bioinformatics Institute, MRC-IEU = Medical Research Council Integrative Epidemiology Unit, MS = multiple sclerosis, RA = rheumatoid arthritis, SLE = systemic lupus erythematosus, TMD = temporomandibular joint disorders.

### 2.3. Selection of the genetic instruments

To determine whether genetic IVs are qualified and meet the 3 MR assumptions, we employed many quality control approaches. First, the IVs for AIDs were selected from SNPs related to AIDs with *P* < 5 × 10^−8^. Second, we use the aggregation program *R*^2^ < 0.001, window size = 10,000 kb, to filter out SNPs that are in a significant linkage imbalance (LD).^[12]^ Furthermore, each SNP’s *F*-statistic is computed using the formula *F* = β^2^/se^2^. An IV will not be included in the MR analysis since it is deemed a weak instrument with an *F*-statistic of <10.^[13]^ Finally, we removed all palindromic SNPs with moderate allele frequencies from the previously selected SNPs to guarantee consistency and correctness of the results.^[14]^

### 2.4. Statistical analysis

In this work, R (Version 4.3.1) was used for all statistical analyses. To determine the causal relationship between AIDs and TMD, MR analysis was performed using the “TwoSampleMR” package in R. The main analysis of this study used inverse variance weighted (IVW) with a significance level of *P* < for .05 to determine plausible causal effects.^[15]^ As supplemental techniques to MR analysis, weighted median, weighted mode, simple mode, and MR-Egger regression were employed to accurately determine the causative impact and mitigate the impacts of horizontal pleiotropy.^[16]^ To account for multiple testing across the 5 autoimmune diseases, we applied Bonferroni correction, resulting in a significance threshold of *P* < .01. Associations with 0.01 ≤ *P* < .05 were regarded as suggestive evidence of a causal relationship.

### 2.5. Heterogeneity and sensitivity analysis

To evaluate heterogeneity, we administered the Cochran’s *Q* test, with heterogeneity defined as *P* < .05. The MR-Egger was performed to evaluate whether there is pleotropy between SNPs as IVs. Pleiotropy is nonexistent in causal analysis if *P* > .05, and its impact may be disregarded.^[16]^ To find any SNPs that might have an influence, we also carried out a sensitivity analysis using the Mendelian Randomization Pleiotropy RESidual Sum and Outlier test global test and leave-one-out, methodically eliminating each SNP 1 at a time during the MR analysis.^[17]^

### 2.6. Bioinformatic analysis

From the GEO database, the RA-related dataset GSE55235 was acquired. (https://www.ncbi.nlm.nih.gov/gds/). One TMD-related dataset is GSE150057. To acquire DEGs, differentiated expression analytics was carried out utilizing the limma program (*P*.adj < .05 and |log_2_ (FC)|>1), and the “ggplot2” package was used to visualize the outcomes. Using a Venn diagram, it was possible to verify whether there were any shared DEGs between the DEGs for RA and TMD. In order to validate the results of the MR study, the acquired DEGs were examined for the Kyoto Encyclopedia of Genes and Genomes (KEGG) and Gene Ontology (GO) enrichment using using “clusterProfiler” and “org.Hs.e.g..db” packages to comprehend DEGs’ principal functions and pathways.

## 3. Results

### 3.1. Results of MR analysis of AIDs on TMD

A total of 54, 21, 24, 42, and 18 SNPs were found with a significant level of *P* < 5 × 10^−8^ and independent inheritance (*R*^2^ < 0.001 and distance > 10,000 kb) in order to investigate the causal effect of 5 AIDs (RA, MS, AS, SLE, psoriasis) on TMD. The *F*-statistics, all of which are >10, demonstrated that there was no slight instrumental bias. A full description of the IVs for AIDs is TMD in Tables S1–S5, Supplemental Digital Content, https://links.lww.com/MD/R8.

**Figure 2.**
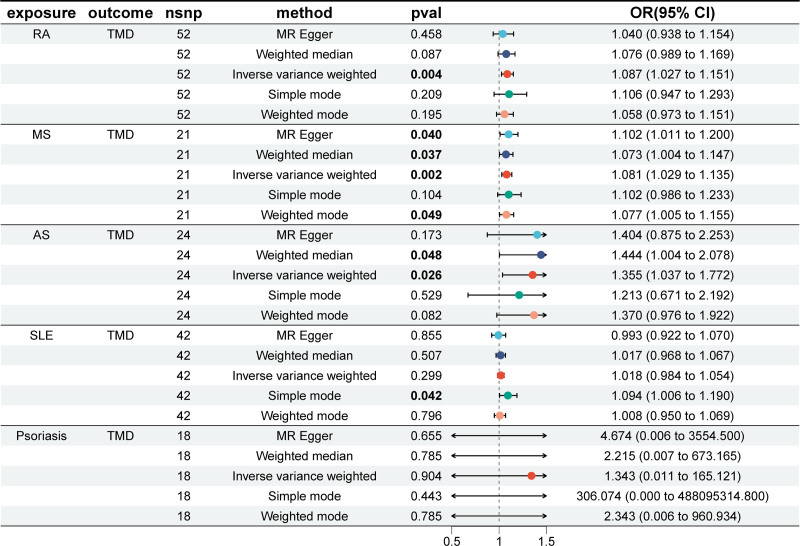
MR estimates for the causal effect of 5 AIDs on TMD. AIDs = autoimmune diseases, AS = ankylosing spondylitis, CI = confidence interval, IVW = inverse variance weighted, MR = Mendelian randomization, MS = multiple sclerosis, OR = odds ratio, RA = rheumatoid arthritis, SLE = systemic lupus erythematosus, SNP = single-nucleotide polymorphism, TMD = temporomandibular joint disorders.

The results of MR analysis (Fig. 2) indicated that RA (IVW: odds ratio [OR] = 1.087; 95% confidence interval [CI] = 1.027–1.151; *P* = .004), MS (IVW: OR = 1.081; 95% CI = 1.029–1.135; *P* = .002) and AS (IVW: OR = 1.355; 95% CI = 1.037–1.772; *P* = .026) increased the risk of TMD. For MS and AS, the weighted median method also showed significant associations (MS: *P* = .037; AS: *P* = .048), consistent with the IVW results, further supporting the robustness of these findings.

In sensitivity analyses, no significant heterogeneity was detected by Cochran’s *Q* test. MR-Egger regression and the Mendelian Randomization Pleiotropy RESidual Sum and Outlier test global test indicated no evidence of directional pleiotropy or outlying SNPs (Table 2). The scatter plots, funnel plots, and leave-one-out analyses are presented in Figures S1 to S3, Supplemental Digital Content, https://links.lww.com/MD/R8, indicating that no abnormal SNPs were found, confirming the reliability of our analysis.

**Table 2:** MR estimates of assessing the bidirectional causal association between TMD and AIDs.

Exposure	Outcome	SNPs	IVW		MR-Egger regression		MR-PRESSO	
			*Q* statisti c	*Q*_*P*val	Intercept	*P*-value	RSSobs	*P*-value
RA	TMD	54	53.356	0.460	0.009	.235	59.915	.500
MS		21	9.799	0.972	−0.007	.602	10.504	.975
AS		5	26.487	0.278	−0.002	.858	28.266	.342
SLE		42	44.955	0.310	0.010	.462	47.312	.400
Psoriasis		18	13.470	0.704	−0.006	.600	18.032	.691
TMD	RA	5	1.761	0.780	0.022	.517		
	MS	4	3.277	0.351	−0.096	.297	5.277	.549
	AS	0	–	–	–	–	–	–
	SLE	4	2.174	0.537	0.100	.345	4.015	.670
	Psoriasis	3	0.623	0.732	−0.001	.575	2.096	.760

AID = autoimmune disease, AS = ankylosing spondylitis, IVW = inverse variance weighted, MR = Mendelian randomization, MR-PRESSO = Mendelian Randomization Pleiotropy RESidual Sum and Outlier test, MS = multiple sclerosis, RA = rheumatoid arthritis, SLE = systemic lupus erythematosus, SNP = single-nucleotide polymorphism, TMD = temporomandibular joint disorders.

After applying Bonferroni correction for multiple testing (5 exposures, significance threshold *P* < .01), the associations of RA and MS with TMD remained significant, while the association for AS was attenuated to a suggestive level (*P* = .026). No significant associations were observed for SLE or psoriasis after correction.

### 3.2. Results of MR analysis of TMD on AIDs

**Figure 3.**
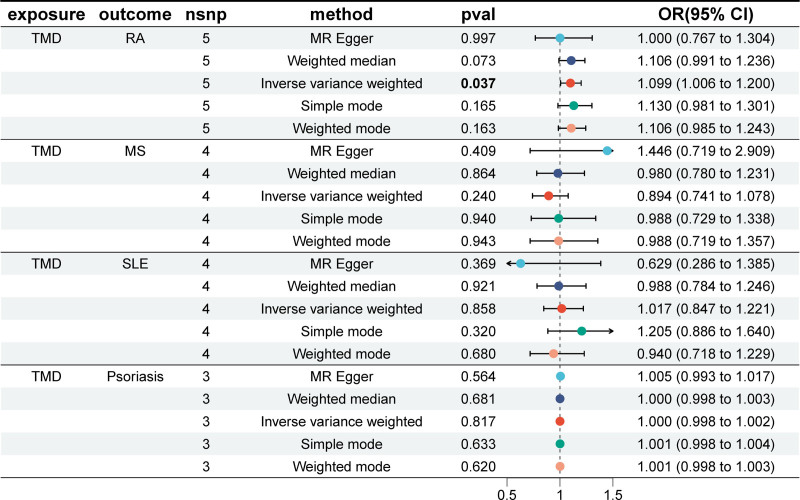
MR estimates for the causal effect of TMD on 5 AIDs. AIDs = autoimmune diseases, AS = ankylosing spondylitis, CI = confidence interval, IVW = inverse variance weighted, MR = Mendelian randomization, MS = multiple sclerosis, OR = odds ratio, RA = rheumatoid arthritis, SLE = systemic lupus erythematosus, SNP = single-nucleotide polymorphism, TMD = temporomandibular joint disorders.

In the reverse study, we explored the causal relationship between TMD and 5 common AIDs. There were 5, 4, 4, 3 SNPs respectively applied to the TMD in the MR Study of RA, MS, SLE and psoriasis (Table S6, Supplemental Digital Content, https://links.lww.com/MD/R8). In addition, no SNPs associated with TMD were found in AS. The results of MR showed that only TMD had a causal relationship with RA (Fig. 3). Sensitivity analyses revealed no evidence of heterogeneity or horizontal pleiotropy, and no single SNP was found to disproportionately influence the results (Table 2). The scatter plots, funnel plots, and leave-one-out analyses are provided in Figures S4 to S6, Supplemental Digital Content, https://links.lww.com/MD/R8 for reference, further supporting the reliability and robustness of our results.

### 3.3. Bioinformatic analysis

**Figure 4.**
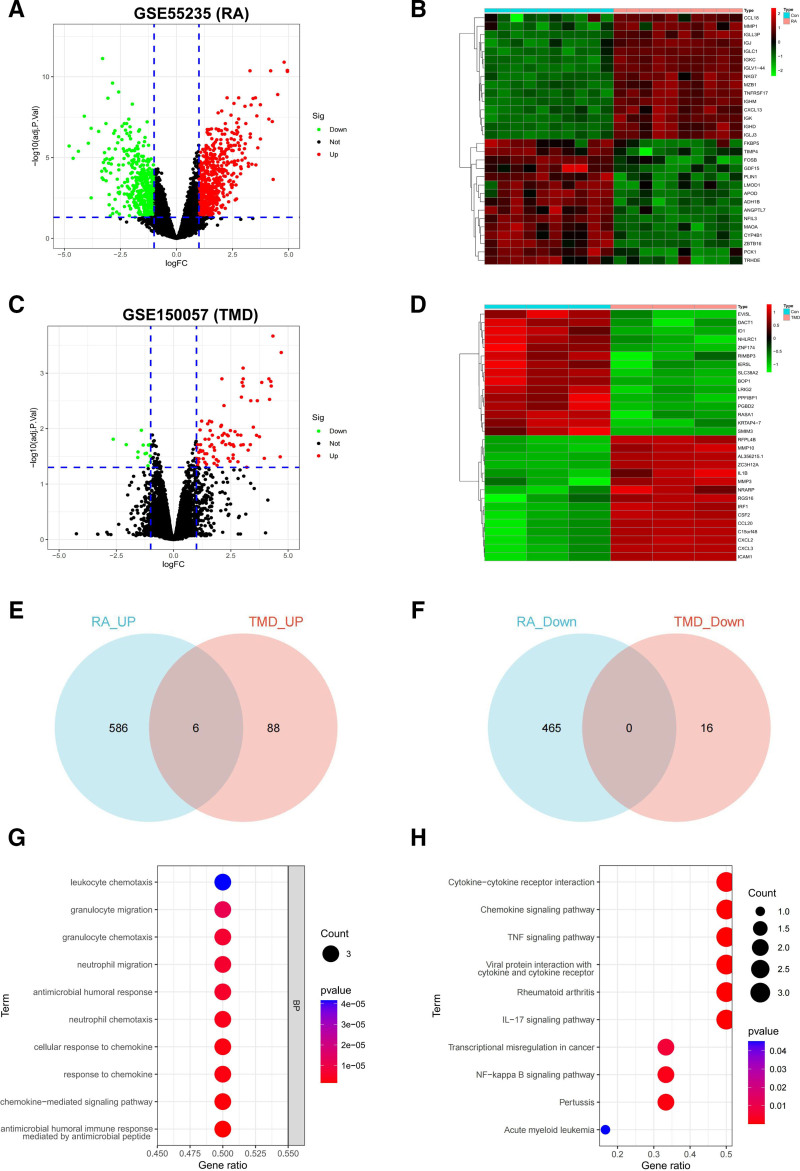
Bioinformatic analysis. (A, B) Volcano map and heat map of RA-associated DEGs. (C, D) Volcano map and heat map of TMD-associated DEGs. (E, F) Venn diagram of DEGs common to RA and TMD. (G, H) GO and KEGG enrichment analysis of DEGs. DEGs = differentially expressed genes, GO = Gene Ontology, KEGG = Kyoto Encyclopedia of Genes and Genomes, RA = rheumatoid arthritis, TMD = temporomandibular disorders.

After analyzing the RA-related dataset GSE55235, 1057 DEGs were obtained, of which 465 were underexpressed and 592 were highly expressed (Fig. 4A, B). For TMD, 110 DEGs were acquired, of which 16 were underexpressed and 94 were highly expressed (Fig. 4C, D). The Venn diagram showed that, in indeed, RA and TMD shared certain DEGs, including COL3A1, CXCL6, BCL2A1, MMP3, CCL13, and CXCL5 (Fig. 4E, F). These results suggest a potential association between TMD and RA. DEGs common to both RA and TMD were found to primarily perform functions such as chemokine – mediated signaling pathway, response to chemokine, neutrophil chemotaxis, granulocyte chemotaxis, and so on. Furthermore, these DEGs participate in pathways such as the chemokine, TNF, NF-β B, and IL-17 signaling pathways (Fig. 4G, H).

## 4. Discussion

To the best of our knowledge, this work is the first to evaluate the causal relationship between AIDs and TMD using MR techniques. The results imply that RA, MS, AS, and TMD have a positive genetic causal link, which means that these conditions are risk factors for TMD. Notably, after Bonferroni correction, RA and MS maintained significant associations with TMD, while AS showed only suggestive evidence. These findings indicate that RA and MS provide the most robust genetic support for a causal relationship with TMD. However, no relationship was found between SLE and psoriasis on TMD. This study design effectively minimizes the effects of potential confounders and reverse causation.

Consistent with our findings, numerous prior research have demonstrated that patients diagnosed with AIDs have a significant increased chance of acquiring TMD. For example, a previous longitudinal follow-up study using demographic information from the 2002 to 2015 Korean National Health Insurance Service Health Examination cohort found that compared to a control group, Hazard ratio (HR) for TMD in RA patients was 2.52 (95% CI = 1.70–3.74).^[7]^ A study in Taiwan tracked 17,317 newly diagnosed RA patients and 17,317 non-RA controls found that TMD episodes were 2.538 times more common in RA patients than in non-RA patients.^[18]^ Similarly, a cross-sectional study of TMD in patients with early rheumatoid arthritis (ERA) and RA revealed a higher relative risk of TMD in RA and ERA patients.^[19]^ Meta-analysis showed that RA was associated with structural bone changes in TMJ diagnosed by cone beam computed tomography (CBCT), and RA patients were more likely to have osteoarthritis variations in this joint.^[20]^ Some cases suggest that timely drug treatment for ERA patients can reduce the prevalence of TMD. It indicates that timely diagnosis and treatment of RA are of great importance to decreasing the prevalence of TMD.^[19]^ In addition, Carvalho et al recruited 120 subjects (60 MS patients and 60 healthy controls) and found that the prevalence of MS patients was 61.7% compared to 18.3% in the control group.^[21]^ A link between MS and TMD was also demonstrated by a meta-analysis of 8 studies (RR 2.10; 95% CI: 1.21–3.65).^[9]^ Huang et al conducted a cohort study involving 3204 patients with AS and 12,816 age-matched and sex-matched comparisons. According to the findings, the AS cohort had a 2.88-fold greater incidence of TMD than the control group. After adjusting for age, sex, and comorbidity, the risk of TMD in the AS cohort was 2.66 times higher (95% CI = 1.79–3.97; *P* < .0001).^[8]^ Moreover, a cross-sectional study involving 30 patients with AS revealed an increased prevalence of TMD illness in AS patients.^[22]^ Despite the results of several observational studies reporting a relationship between SLE and psoriasis and TMD, our findings did not find an association between these diseases.^[23,24]^ This contradiction may be explained by the observational research methodology’s bias susceptibility to small sample sizes, unavoidable confounders, and reverse causality. As a result, we conducted these 2-sample MR Analyses and obtained reliable and robust results showing no causal relationship between SLE and psoriasis on TMD susceptibility.

Our study supported a bidirectional causal relationship between RA and TMD. To further explore the molecular basis of this association, we conducted bioinformatics analysis. The Venn diagram showed that TMD and RA shared DEGs, highlighting common molecular features and providing clues for further mechanistic investigation. For example, the expression of COL3A1, a gene crucial to cartilage function, has been discovered in association with the radiographic level of osteoarthritis (OA).^[25]^ According to research, COL3A1 may be an important gene signature that interacts with extracellular matrix receptors and promotes focal adhesion, which may contribute to the development of osteoarthritis.^[26]^ CXCL5 and 6 are 2 of the main members of the CXC subfamily of chemokines, which are related to the invasion and migration of various cancers. Studies have also shown that CXCL6 expression is raised in RA patients, and it may be involved in neutrophil migration and angiogenesis.^[27]^ Studies have suggested that IL-17-mediated articular angiogenesis may be due in part to CXCL5 induction.^[28]^ BCL2A1 is an antiapoptotic BCL2 family member that does not affect cell cycle progression. BCL2A1 may control CD4+ cell activation and differentiation into pro-inflammatory TH17 cells, and BCL2A1 is a potential target for controlling autoimmune/inflammatory diseases.^[29]^ MMP-3 is a significant contributor to joint conditions such as TMJ disease and rheumatism. MMP-3 contributes to the deterioration of joints in RA patients by breaking down several types of collagen, fibronectin, proteoglycan, elastin, and laminin.^[30]^ In inflammatory tissues, CCL13 is a key molecule associated with the selected recruitment of cell lineages and their subsequent activation. It is implicated in numerous chronic inflammatory illnesses.^[31]^ The DEGs common to RA and TMD were primarily involved in chemokine – mediated signaling pathway, response to chemokine, neutrophil chemotaxis, granulocyte chemotaxis, and so on, according to GO analysis. Furthermore, these DEGs are engaged in a number of pathways, including the TNF signaling pathway, chemokine signaling pathway, NF-β B signaling pathway, and IL-17 signaling pathways, suggesting potential molecular mechanisms underlying the observed association in the MR analysis.

It is unclear what factors contribute to the association between RA and TMD. Previous research has suggested that immunological diseases could play a role in the development of chronic TMJ pain. A large number of inflammatory cells infiltrate the synovial microenvironment in RA patients, including B lymphocytes, antigen-presenting cells, T lymphocytes, and fibroblast-like synovial cells (FLSs). The TLRs pathway expressed on FLSs can be stimulated to produce pro-inflammatory factors, including TNF-α, IL-1, IL-6, IL-17 and IL-23. They act on synovial cells and osteoblasts, triggering synovial changes that lead to synovitis and persistent local inflammation of the joints, which may be related to the development of TMD.^[32–34]^ At present, the pathological explanation for the association between RA and TMD is that the surface of the medial condylar joint in RA patients is covered with inflammatory granulation, resulting in the destruction of the bone structure in the joint area.^[35]^ Articular cartilage deterioration, accompanied by proteolytic enzymes and activation of degrading proteoglycans in synovial fluid, may cause secondary inflammatory responses accompanied by joint component degradation, including the TMJ.^[36]^ This inflammatory reaction in RA patients might impair movement in the TMJ area and create minor edema.^[37]^ Everything above confirms the MR analysis results in this investigation.

Although observational studies have been extensively utilized to initially determine causal factors, their credibility is frequently limited by the inevitable occurrence of reverse causation and potential confounding factors. When it comes to determining causal inference in clinical research, RCTs are considered the gold standard. The time-consuming nature of extensive follow-up and the associated high costs, however, make them frequently difficult to execute. The MR methodology provides a solution to the previously mentioned problems by using genetic variants as IVs. Firstly, confounding variables are efficiently avoided by distributing genetic variation randomly during conception.^[38]^ Secondly, the distribution of genotypes occurs before exposure in time, guaranteeing that the association between genotype and disease is unaffected by reverse causality. Lastly, exposure factors derived from a genetic perspective frequently last a lifetime, which mitigates the attenuation bias (regression dilution bias) in comparison to the instantaneous results acquired from RCTs.^[39]^

Our study has several limitations. First, because all GWAS datasets were derived from individuals of European ancestry, the generalizability of our findings to non-European populations remains uncertain. Future studies incorporating East Asian and other ancestries are needed to validate whether these associations hold across diverse genetic backgrounds. Second, the number of genome-wide significant SNPs available as IVs for AS and psoriasis was relatively small, which may have reduced the statistical power of our MR analyses for these traits. Consequently, the null finding for psoriasis should be interpreted with caution, and larger GWAS datasets will be required for confirmation. Third, potential sample overlap exists because both TMD and several autoimmune diseases were partly derived from FinnGen, which could introduce bias in the causal estimates. Future studies using completely independent cohorts will be necessary to rule out the influence of sample overlap. In addition, potential selection bias in the GWAS cohorts cannot be excluded, as TMD cases were clinically diagnosed while controls were drawn from population-based cohorts. Such differences in case and control definition may have influenced the effect estimates. Future studies using uniformly defined case–control cohorts or prospective population-based designs will be needed to minimize this bias. Finally, although MR analysis reduces the risk of confounding and reverse causation, the possibility of residual horizontal pleiotropy cannot be completely excluded.

## 5. Conclusion

Based on the study, there is a considerable increase in the likelihood of TMD development in patients with RA, MS, and AS. It emphasizes the necessity of keeping an eye on temporomandibular function in individuals with RA, MS, and AS as well as the necessity of future study into the mechanisms behind the relationship between these illness.

## Acknowledgments

The authors sincerely thank the researchers and the participants of the original GWAS for the collection and management of the large-scale data resources.

## Author contributions

**Data curation:** Xiaorong Yan

**Formal analysis:** Feihan Gu, Wenkai Huang

**Funding acquisition:** Yuanyin Wang

**Investigation:** Weimin Bao

**Methodology:** Erli Wu

**Resources:** Mingyu Zhao

**Supervision:** Hu Zheng

**Visualization:** Ran Chen

**Writing – original draft:** Feihan Gu, Weimin Bao, Mingyu Zhao, Wenkai Huang, Erli Wu, Xiaorong Yan, Hu Zheng

**Writing – review & editing:** Yuanyin Wang, Ran Chen

## Supplementary Material


