# Supplementary material for: The relationship between autoimmune diseases and temporomandibular disorders: A study combined with the GEO database
Source: Medicine (Baltimore). 2025 Dec 26;104(52):e46760. doi: 10.1097/MD.0000000000046760 (PMC12747028; doi:10.1097/MD.0000000000046760)
Supplement: Supplementary file 1 [file medi-104-e46760-s001.docx]

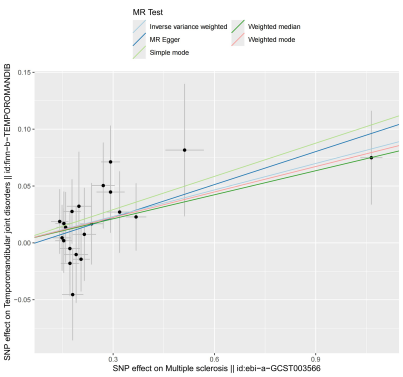

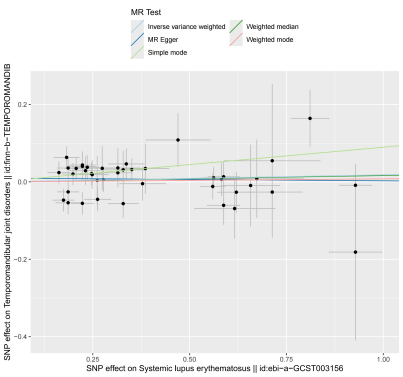

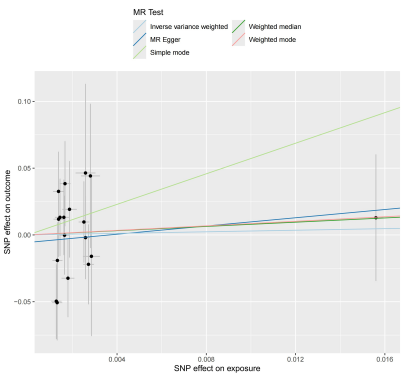

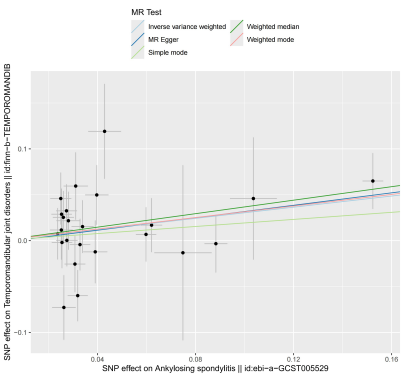

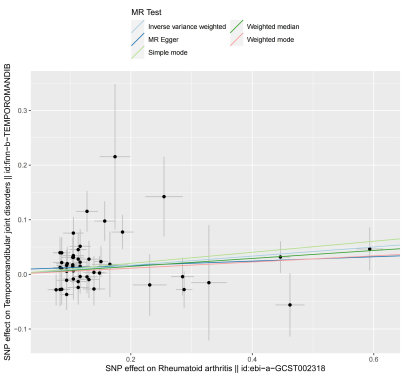


A

B

C

D

E

**Supplementary Figure 1**. Scatter plots of MR of five AIDs on TMD. (A) RA on TMD; (B) MS on TMD; (C) AS on TMD; (D) SLE on TMD; (E) Psoriasis on TMD; Abbreviations: MR Mendelian randomization; AIDs, autoimmune diseases; TMD, temporomandibular joint disorders; RA, rheumatoid arthritis; MS, multiple sclerosis; AS, ankylosing spondylitis; SLE, systemic lupus erythematosus.


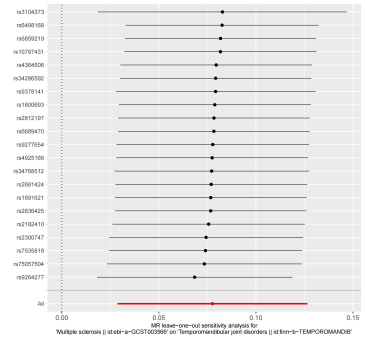

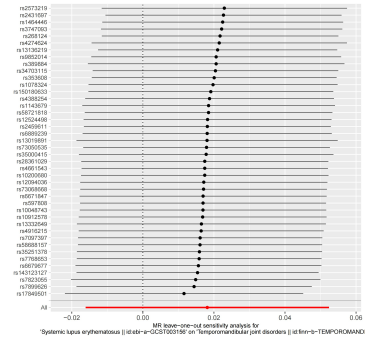

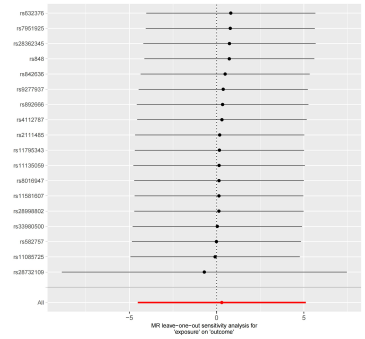

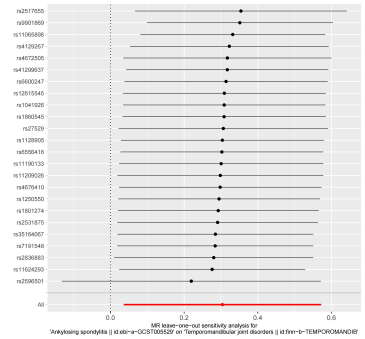

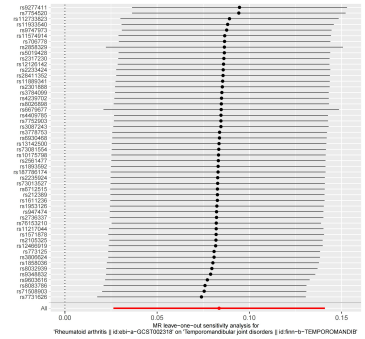


A

B

C

D

E

**Supplementary Figure 2**. Leave-one-out plot of MR of five AIDs on TMD. (A) RA on TMD; (B) MS on TMD; (C) AS on TMD; (D) SLE on TMD; (E) Psoriasis on TMD; Abbreviations: MR Mendelian randomization; AIDs, autoimmune diseases; TMD, temporomandibular joint disorders; RA, rheumatoid arthritis; MS, multiple sclerosis; AS, ankylosing spondylitis; SLE, systemic lupus erythematosus.


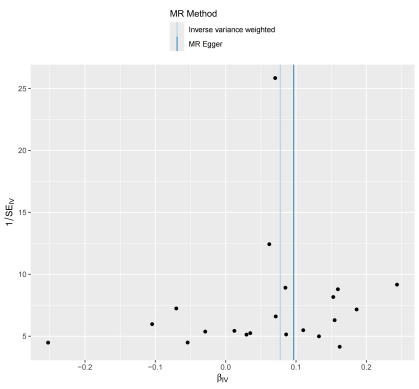

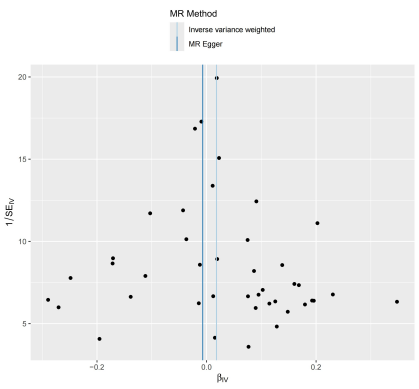

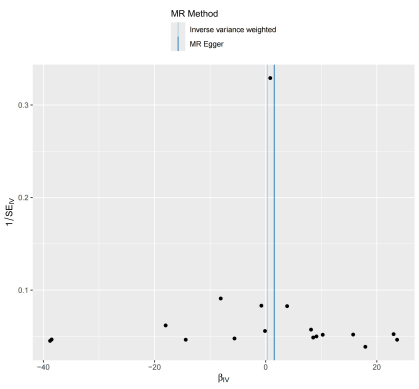

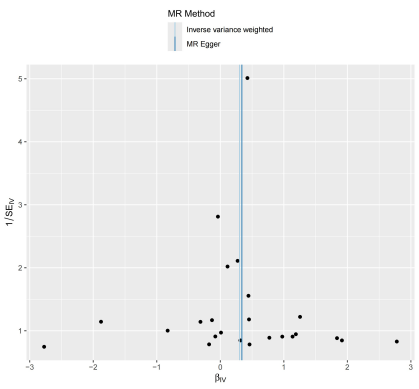

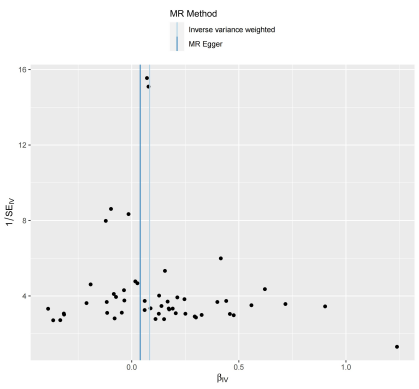


A

B

C

D

E

**Supplementary Figure 3.** Funnel plot of MR analyses of five AIDs on TMD. (A) RA on TMD; (B) MS on TMD; (C) AS on TMD; (D) SLE on TMD; (E) Psoriasis on TMD; Abbreviations: MR Mendelian randomization; AIDs, autoimmune diseases; TMD, temporomandibular joint disorders; RA, rheumatoid arthritis; MS, multiple sclerosis; AS, ankylosing spondylitis; SLE, systemic lupus erythematosus.


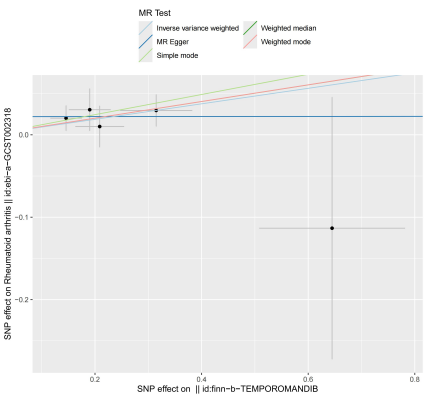

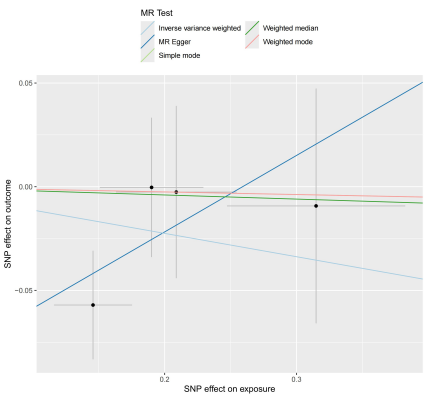

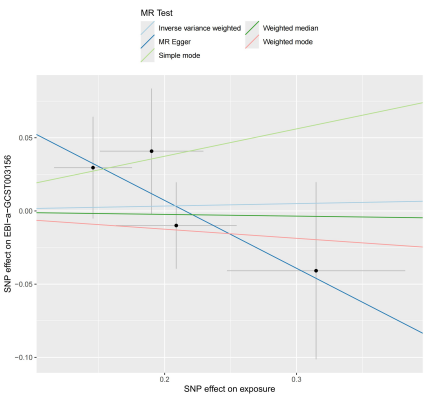

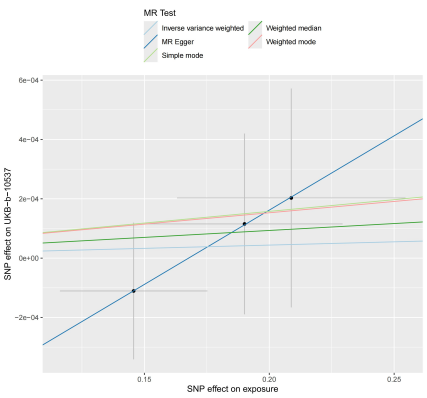


A

B

C

D

**Supplementary Figure 4.** Scatter plots of MR of TMD on AIDs. (A) TMD on RA; (B) TMD on MS; (C) TMD on SLE; (D) TMD on Psoriasisi; Abbreviations: MR Mendelian randomization; AIDs, autoimmune diseases.


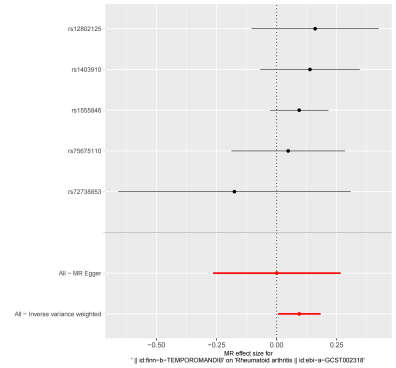

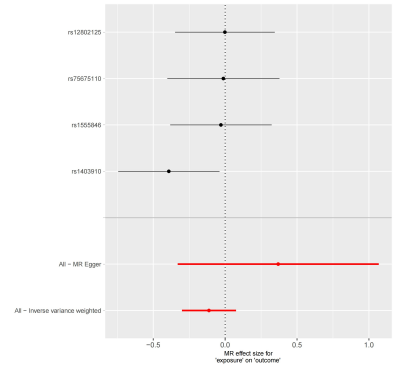

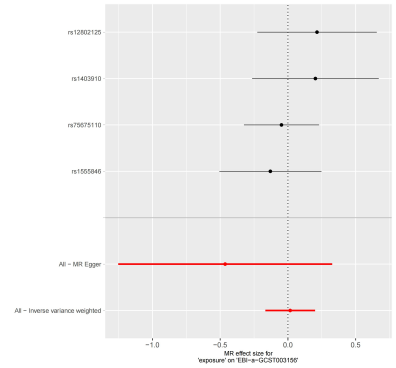

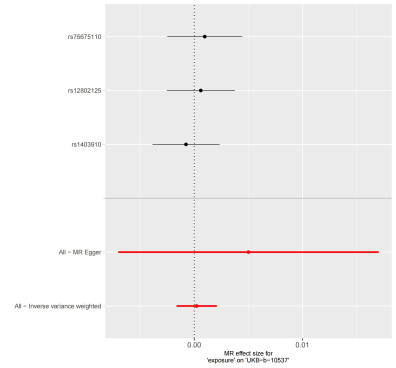


A

B

C

D

**Supplementary Figure 5**. Leave-one-out plot of MR of TMD on AIDs. (A) TMD on RA; (B) TMD on MS; (C) TMD on SLE; (D) TMD on Psoriasisi; Abbreviations: MR Mendelian randomization; AIDs, autoimmune diseases; TMD, temporomandibular joint disorders; RA, rheumatoid arthritis; MS, multiple sclerosis; SLE, systemic lupus erythematosus.


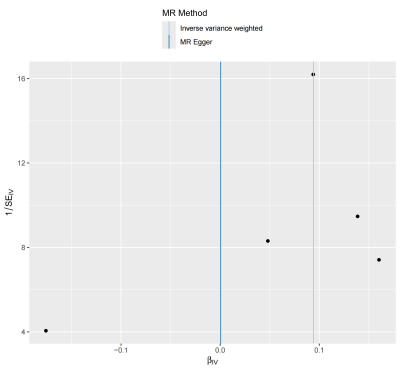

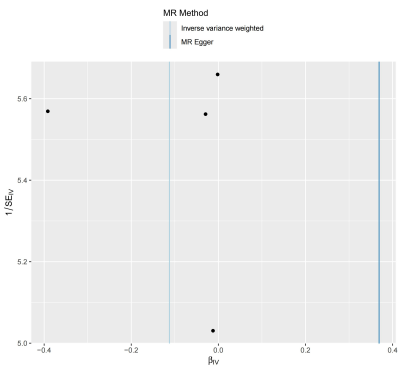

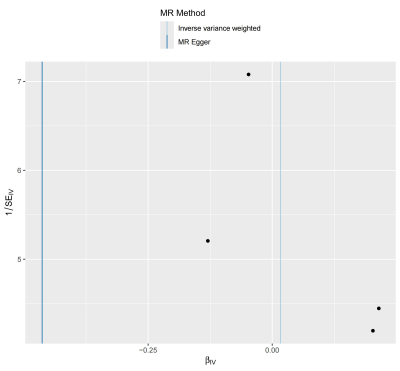

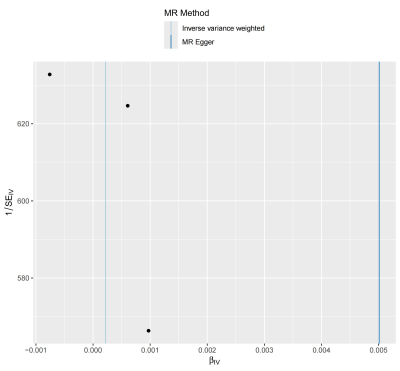


A

B

C

D

**Supplementary Figure 6.** Funnel plot of MR analyses of TMD on AIDs. (A) TMD on RA; (B) TMD on MS; (C) TMD on SLE; (D) TMD on Psoriasisi; Abbreviations: MR Mendelian randomization; AIDs, autoimmune diseases; TMD, temporomandibular joint disorders; RA, rheumatoid arthritis; MS, multiple sclerosis; SLE, systemic lupus erythematosus.

**Supplementary Table 1.** Detailed information of instrumental variables used in the Mendelian randomization analysis of RA on TMD (significant level of p < 5e-08). Abbreviations: TMD, temporomandibular joint disorders; RA, rheumatoid arthritis.

| **SNP** | **effect_allele** | **other_allele** | **F-statistic** | **associated with RA** | | | **associated with TMD** | | |
| --- | --- | --- | --- | --- | --- | --- | --- | --- | --- |
|  |  |  |  | **beta** | **se** | **pval** | **beta** | **se** | **pval** |
| rs10175798 | A | G | 38.70200639 | 0.0861777 | 0.0138525 | 5.39995E-09 | 0.0861777 | 0.0109 | 0.6995 |
| rs11217044 | C | T | 51.69691671 | -0.131028 | 0.0182235 | 3.59998E-15 | -0.131028 | -0.0279 | 0.4034 |
| rs112733823 | T | C | 229.395654 | 0.285179 | 0.0188289 | 6.59933E-39 | 0.285179 | -0.0042 | 0.9025 |
| rs11574914 | A | G | 40.06789519 | 0.113329 | 0.0179037 | 2.09991E-13 | 0.113329 | -0.0239 | 0.4455 |
| rs11889341 | T | C | 55.45614566 | 0.131028 | 0.017595 | 6.70039E-19 | 0.131028 | -0.0096 | 0.771401 |
| rs11933540 | C | T | 60.50338484 | 0.139262 | 0.0179037 | 8.80035E-17 | 0.139262 | -0.0267 | 0.3762 |
| rs12126142 | A | G | 25.93929681 | -0.0833816 | 0.0163716 | 3.50002E-09 | -0.0833816 | 0.0278 | 0.3651 |
| rs12466919 | T | C | 70.61387432 | 0.113329 | 0.0134864 | 5.70033E-13 | 0.113329 | 0.0279 | 0.3451 |
| rs13142500 | C | T | 30.40584177 | 0.0943107 | 0.0171034 | 0.000000005 | 0.0943107 | 0.0083 | 0.766999 |
| rs1571878 | T | C | 165.34496 | -0.150823 | 0.0117293 | 6.09958E-30 | -0.150823 | -0.0234 | 0.408 |
| rs1611236 | A | G | 105.6353851 | -0.116534 | 0.0113383 | 2.09991E-15 | -0.116534 | -0.0223 | 0.5241 |
| rs1858036 | G | A | 66.93076603 | -0.113329 | 0.0138525 | 1.20005E-14 | -0.113329 | -0.0453 | 0.141 |
| rs187786174 | A | G | 47.46703602 | -0.116534 | 0.0169144 | 3.29989E-14 | -0.116534 | -0.0149 | 0.6075 |
| rs1893592 | C | A | 55.73265308 | -0.10436 | 0.0139791 | 3.69999E-12 | -0.10436 | -0.0145 | 0.6303 |
| rs1953126 | C | T | 36.62845648 | -0.0861777 | 0.0142392 | 0.000000001 | -0.0861777 | -0.0216 | 0.4439 |
| rs2105325 | C | A | 37.10507058 | 0.105361 | 0.0172967 | 3.09999E-10 | 0.105361 | 0.0344 | 0.3276 |
| rs212389 | A | G | 27.35364331 | 0.0953102 | 0.0182235 | 3.29997E-10 | 0.0953102 | 0.0196 | 0.5269 |
| rs2233424 | T | C | 70.19231546 | 0.231112 | 0.0275853 | 7.59976E-19 | 0.231112 | -0.0193 | 0.7317 |
| rs2235924 | A | G | 32.47875845 | -0.0943107 | 0.0165486 | 3.59998E-09 | -0.0943107 | -0.0165 | 0.5648 |
| rs2301888 | A | G | 55.86258863 | -0.127833 | 0.0171034 | 2.19989E-18 | -0.127833 | 0.0046 | 0.8762 |
| rs2317230 | T | G | 30.30979374 | 0.076961 | 0.0139791 | 0.000000021 | 0.076961 | -0.0282 | 0.3204 |
| rs2561477 | A | G | 57.74592653 | -0.0833816 | 0.0109726 | 1.89998E-09 | -0.0833816 | -0.0126 | 0.6762 |
| rs2736337 | C | T | 84.44202107 | 0.105361 | 0.0114657 | 4.79954E-12 | 0.105361 | 0.031 | 0.3922 |
| rs28411352 | T | C | 70.61387432 | 0.113329 | 0.0134864 | 3.59998E-12 | 0.113329 | -0.0132 | 0.6692 |
| rs2858329 | G | A | 759.0835791 | 0.446287 | 0.0161983 | 1E-200 | 0.446287 | 0.0318 | 0.2683 |
| rs3087243 | A | G | 64.82443647 | -0.139262 | 0.0172967 | 1.69981E-22 | -0.139262 | -0.0037 | 0.9003 |
| rs3778753 | G | A | 37.10507058 | 0.105361 | 0.0172967 | 1.10002E-14 | 0.105361 | 0.0064 | 0.8195 |
| rs3784099 | A | G | 32.47875845 | -0.0943107 | 0.0165486 | 7.10003E-10 | -0.0943107 | 0.0108 | 0.723299 |
| rs3806624 | G | A | 55.28948982 | 0.0833816 | 0.0112137 | 1.89998E-08 | 0.0833816 | 0.0397 | 0.1568 |
| rs4239702 | C | T | 100.9924468 | 0.116534 | 0.011596 | 8.99912E-15 | 0.116534 | -0.004 | 0.8961 |
| rs4409785 | C | T | 37.10507058 | 0.105361 | 0.0172967 | 2.99999E-08 | 0.105361 | -0.0084 | 0.8226 |
| rs5019428 | A | G | 38.70200639 | 0.0861777 | 0.0138525 | 7.19996E-10 | 0.0861777 | -0.0272 | 0.343 |
| rs6679677 | A | C | 722.9848338 | 0.593327 | 0.0220663 | 2.0989E-149 | 0.593327 | 0.0465 | 0.2372 |
| rs6712515 | C | T | 55.73265308 | -0.10436 | 0.0139791 | 6.70039E-15 | -0.10436 | -0.0175 | 0.5352 |
| rs6930468 | G | A | 69.18724683 | 0.0943107 | 0.0113383 | 5.50047E-11 | 0.0943107 | 0.0057 | 0.845 |
| rs706778 | T | C | 38.70200639 | 0.0861777 | 0.0138525 | 1.5E-10 | 0.0861777 | -0.0273 | 0.3318 |
| rs71508903 | T | C | 83.79678323 | 0.157004 | 0.0171513 | 2.29985E-20 | 0.157004 | 0.0976 | 0.00673395 |
| rs73013527 | T | C | 32.47875845 | -0.0943107 | 0.0165486 | 9.79941E-11 | -0.0943107 | -0.0163 | 0.565501 |
| rs73081554 | T | C | 31.68823116 | 0.165514 | 0.0294026 | 4.60002E-08 | 0.165514 | 0.0184 | 0.7573 |
| rs76153210 | T | C | 48.04152266 | 0.173953 | 0.0250971 | 2.19999E-09 | 0.173953 | 0.2154 | 0.1061 |
| rs773125 | G | A | 36.62845648 | -0.0861777 | 0.0142392 | 4.39997E-10 | -0.0861777 | -0.0395 | 0.1631 |
| rs7731626 | A | G | 105.7926091 | -0.18633 | 0.0181157 | 7.29962E-24 | -0.18633 | -0.0775 | 0.01277 |
| rs7752903 | G | T | 126.8912923 | 0.328504 | 0.0291625 | 2.70023E-26 | 0.328504 | -0.0152 | 0.8855 |
| rs7754520 | T | C | 378.9503019 | -0.462035 | 0.0237347 | 8.99912E-67 | -0.462035 | 0.0559 | 0.3342 |
| rs8026898 | A | G | 129.8023912 | 0.14842 | 0.0130272 | 6.4998E-19 | 0.14842 | 0.0025 | 0.9359 |
| rs8032939 | C | T | 44.37181597 | 0.116534 | 0.0174944 | 4.79954E-16 | 0.116534 | 0.0514 | 0.0988098 |
| rs8083786 | G | A | 52.17967682 | 0.127833 | 0.0176967 | 1E-15 | 0.127833 | 0.1154 | 0.00184999 |
| rs9277411 | T | C | 459.0441561 | -0.287682 | 0.0134272 | 1.50003E-85 | -0.287682 | 0.0278 | 0.4048 |
| rs9348832 | A | G | 68.80725792 | 0.254642 | 0.0306982 | 5.19996E-19 | 0.254642 | 0.1423 | 0.0499298 |
| rs947474 | A | G | 33.38311413 | 0.10436 | 0.0180622 | 0.000000015 | 0.10436 | 0.0313 | 0.3909 |
| rs9603616 | T | C | 39.66375669 | -0.105361 | 0.0167295 | 4.60045E-12 | -0.105361 | -0.0756 | 0.0105099 |
| rs9747973 | T | C | 72.29660049 | -0.0943107 | 0.0110918 | 1.9002E-12 | -0.0943107 | 0.0368 | 0.1951 |

**Supplementary Table 2**. Detailed information of instrumental variables used in the Mendelian randomization analysis of MS on TMD (significant level of p < 5e-08). Abbreviations: TMD, temporomandibular joint disorders; MS, multiple sclerosis.

| **SNP** | **effect_allele** | **other_allele** | **F-statistic** | **associated with MS** | | | **associated with TMD** | | |
| --- | --- | --- | --- | --- | --- | --- | --- | --- | --- |
|  |  |  |  | **beta** | **se** | **pval** | **beta** | **se** | **pval** |
| rs10797431 | T | G | 40.66093982 | 0.172161 | 0.0269989 | 1.81E-10 | -0.018 | 0.0288 | 0.5327 |
| rs1800693 | C | T | 37.21540569 | -0.153794 | 0.0252103 | 1.06E-09 | -0.0019 | 0.0283 | 0.9461 |
| rs1891621 | G | A | 30.74662195 | 0.141813 | 0.0255751 | 2.94E-08 | 0.0188 | 0.0284 | 0.5092 |
| rs2182410 | C | T | 46.05830215 | -0.178246 | 0.0262643 | 1.15E-11 | -0.0276 | 0.0283 | 0.33 |
| rs2300747 | G | A | 49.76232304 | 0.292332 | 0.0414406 | 1.74E-12 | 0.0447 | 0.0358 | 0.2119 |
| rs2681424 | C | T | 37.42284718 | 0.154189 | 0.0252049 | 9.51E-10 | 0.017 | 0.0281 | 0.5441 |
| rs2812197 | T | C | 32.85177413 | 0.148801 | 0.0259613 | 9.95E-09 | 0.0044 | 0.029 | 0.8804 |
| rs2836425 | T | C | 30.81207046 | -0.198692 | 0.0357948 | 2.84E-08 | -0.0322 | 0.0479 | 0.501 |
| rs3104373 | C | T | 1069.590453 | 1.06477 | 0.0325572 | 1.00E-200 | 0.0749 | 0.0412 | 0.06952 |
| rs34286592 | T | C | 29.88545256 | -0.190516 | 0.0348499 | 4.58E-08 | 0.0103 | 0.0424 | 0.8084 |
| rs34768512 | A | G | 69.15023436 | 0.319284 | 0.0383955 | 9.12E-17 | 0.0271 | 0.0358 | 0.4491 |
| rs4364506 | A | G | 34.59530786 | 0.171992 | 0.0292415 | 4.06E-09 | -0.005 | 0.032 | 0.8757 |
| rs4925166 | G | T | 35.39893668 | -0.159449 | 0.0267995 | 2.69E-09 | -0.0137 | 0.031 | 0.659699 |
| rs6498168 | G | T | 63.08351162 | 0.205032 | 0.0258145 | 1.98E-15 | -0.0144 | 0.0283 | 0.6125 |
| rs6689470 | A | G | 39.14590974 | -0.214717 | 0.0343181 | 3.93E-10 | -0.0075 | 0.0409 | 0.8551 |
| rs6859219 | A | C | 33.25996125 | 0.180255 | 0.0312555 | 8.06E-09 | -0.0455 | 0.0402 | 0.258 |
| rs75057504 | C | T | 81.20575984 | 0.511711 | 0.0567847 | 2.03E-19 | 0.0816 | 0.0582 | 0.1612 |
| rs7535818 | A | G | 63.62499088 | -0.270772 | 0.0339461 | 1.51E-15 | -0.0504 | 0.0378 | 0.1821 |
| rs9264277 | C | T | 111.2843907 | 0.292399 | 0.0277178 | 5.13E-26 | 0.0712 | 0.0319 | 0.02551 |
| rs9277654 | G | T | 48.48610695 | 0.236216 | 0.0339235 | 3.33E-12 | 0.0168 | 0.0358 | 0.6389 |
| rs9378141 | C | A | 150.7205135 | 0.367939 | 0.0299702 | 1.21E-34 | 0.0228 | 0.0296 | 0.4422 |

**Supplementary Table 3**. Detailed information of instrumental variables used in the Mendelian randomization analysis of AS on TMD (significant level of p < 5e-08). Abbreviations: TMD, temporomandibular joint disorders; AS, ankylosing spondylitis.

| **SNP** | **effect_allele** | **other_allele** | **F-statistic** | **associated with AS** | | | **associated with TMD** | | |
| --- | --- | --- | --- | --- | --- | --- | --- | --- | --- |
|  |  |  |  | **beta** | **se** | **pval** | **beta** | **se** | **pval** |
| rs1041926 | A | G | 40.96172978 | -0.0748291 | 0.0116918 | 1.55E-10 | 0.0132 | 0.0954 | 0.8895 |
| rs11065898 | T | C | 29.83296349 | 0.0262524 | 0.00480641 | 4.71E-08 | -0.0728 | 0.0352 | 0.0385496 |
| rs11190133 | T | C | 56.79210306 | -0.0338671 | 0.00449401 | 4.84E-14 | -0.0153 | 0.0287 | 0.593999 |
| rs11209026 | A | G | 117.7743674 | -0.103584 | 0.00954481 | 1.94E-27 | -0.0459 | 0.0666 | 0.4905 |
| rs1128905 | C | T | 33.54905971 | -0.0237165 | 0.00409459 | 6.95E-09 | -0.0075 | 0.028 | 0.787501 |
| rs11624293 | C | T | 41.03960526 | 0.0428679 | 0.00669161 | 1.49E-10 | 0.1191 | 0.0517 | 0.0211802 |
| rs1250550 | A | C | 36.5824539 | -0.026036 | 0.00430465 | 1.46E-09 | -0.0254 | 0.0287 | 0.3749 |
| rs12615545 | C | T | 37.26600201 | 0.0254728 | 0.00417273 | 1.03E-09 | -0.002 | 0.028 | 0.9445 |
| rs1801274 | G | A | 36.74091877 | 0.0253178 | 0.00417687 | 1.35E-09 | 0.0288 | 0.0279 | 0.3024 |
| rs1860545 | A | G | 39.82087767 | -0.027474 | 0.00435378 | 2.78E-10 | -3.00E-04 | 0.0283 | 0.9927 |
| rs2517655 | T | C | 359.5840685 | 0.0882856 | 0.00465575 | 3.47E-80 | -0.0033 | 0.0314 | 0.9152 |
| rs2531875 | T | G | 41.43683945 | -0.0273233 | 0.00424463 | 1.22E-10 | -0.0325 | 0.0289 | 0.2607 |
| rs2596501 | T | C | 1319.307928 | -0.152336 | 0.00419401 | 1.00E-200 | -0.065 | 0.0304 | 0.0323899 |
| rs27529 | G | A | 208.270196 | -0.0620354 | 0.00429859 | 3.28E-47 | -0.0169 | 0.0294 | 0.5648 |
| rs2836883 | A | G | 69.83216632 | -0.0396768 | 0.00474798 | 6.46E-17 | -0.0498 | 0.0325 | 0.1251 |
| rs35164067 | A | G | 39.41475927 | -0.031078 | 0.00495021 | 3.43E-10 | -0.0595 | 0.0367 | 0.1052 |
| rs4129267 | T | C | 53.00824023 | -0.0307685 | 0.00422605 | 3.32E-13 | 0.0255 | 0.0307 | 0.406 |
| rs41299637 | G | T | 63.25861617 | -0.0390527 | 0.00491011 | 1.81E-15 | 0.0122 | 0.0342 | 0.722099 |
| rs4672505 | G | A | 207.3744313 | -0.0597772 | 0.00415105 | 5.14E-47 | -0.0068 | 0.0296 | 0.8187 |
| rs4676410 | A | G | 32.86114726 | 0.0281014 | 0.00490215 | 9.90E-09 | 0.0217 | 0.0316 | 0.4926 |
| rs6556416 | C | A | 30.04494457 | 0.0252152 | 0.0046002 | 4.22E-08 | 0.0116 | 0.0322 | 0.7191 |
| rs6600247 | C | T | 62.56710603 | 0.0328332 | 0.00415088 | 2.58E-15 | -0.0043 | 0.0281 | 0.8771 |
| rs7191548 | C | T | 34.00021582 | 0.0249853 | 0.00428493 | 5.51E-09 | 0.0459 | 0.0283 | 0.1049 |
| rs9901869 | A | G | 60.88986422 | 0.0319036 | 0.00408853 | 6.04E-15 | -0.0599 | 0.0279 | 0.0320302 |

**Supplementary Table 4**. Detailed information of instrumental variables used in the Mendelian randomization analysis of SLE on TMD (significant level of p < 5e-08). Abbreviations: TMD, temporomandibular joint disorders; SLE, systemic lupus erythematosus.

| **SNP** | **effect_allele** | **other_allele** | **F-statistic** | **associated with SLE** | | | **associated with TMD** | | |
| --- | --- | --- | --- | --- | --- | --- | --- | --- | --- |
|  |  |  |  | **beta** | **se** | **pval** | **beta** | **se** | **pval** |
| rs10048743 | T | G | 31.45810134 | -0.231112 | 0.0412056 | 2.04E-08 | -0.029 | 0.0364 | 0.4255 |
| rs10200680 | T | C | 34.20391984 | -0.248461 | 0.0424835 | 4.96E-09 | -0.0188 | 0.0373 | 0.6141 |
| rs1078324 | A | C | 83.28446466 | -0.71335 | 0.0781665 | 7.11E-20 | 0.0263 | 0.0704 | 0.7083 |
| rs10912578 | G | A | 63.44652606 | -0.24686 | 0.0309918 | 1.65E-15 | -0.0214 | 0.0301 | 0.477 |
| rs1143679 | A | G | 212.0016865 | 0.582216 | 0.0399866 | 5.03E-48 | 0.0065 | 0.0435 | 0.8805 |
| rs12094036 | C | T | 32.23531894 | -0.328504 | 0.0578595 | 1.37E-08 | -0.0312 | 0.0486 | 0.5205 |
| rs12524498 | T | G | 31.0736124 | -0.673345 | 0.120793 | 2.48E-08 | -0.0082 | 0.101 | 0.9357 |
| rs13019891 | T | G | 374.8476147 | -0.562119 | 0.0290336 | 1.65E-83 | -0.0105 | 0.0282 | 0.7094 |
| rs13136219 | T | C | 39.37091669 | -0.174353 | 0.027787 | 3.50E-10 | 0.0471 | 0.0291 | 0.105 |
| rs13332649 | G | A | 70.17473145 | -0.314711 | 0.0375683 | 5.43E-17 | -0.0236 | 0.0312 | 0.4494 |
| rs143123127 | A | G | 31.28174476 | 0.470004 | 0.0840342 | 2.23E-08 | 0.1084 | 0.0694 | 0.118 |
| rs1464446 | T | G | 66.94477944 | -0.328504 | 0.0401497 | 2.79E-16 | 0.0561 | 0.0366 | 0.1248 |
| rs150180633 | T | C | 181.1925808 | 0.928219 | 0.0689573 | 2.66E-41 | -0.1814 | 0.228 | 0.4262 |
| rs17849501 | T | C | 264.4776777 | 0.81093 | 0.0498642 | 1.81E-59 | 0.1642 | 0.073 | 0.0245397 |
| rs2431697 | C | T | 58.01521611 | -0.223144 | 0.0292964 | 2.60E-14 | 0.0554 | 0.0287 | 0.0536204 |
| rs2459611 | T | C | 33.36980543 | 0.261365 | 0.045245 | 7.62E-09 | 0.004 | 0.0631 | 0.9489 |
| rs2573219 | C | A | 187.4711151 | 0.587787 | 0.0429292 | 1.13E-42 | -0.0605 | 0.0502 | 0.2275 |
| rs268124 | T | C | 33.13386659 | 0.18633 | 0.0323703 | 8.60E-09 | -0.0539 | 0.0289 | 0.0618102 |
| rs28361029 | A | G | 39.50365995 | -0.385662 | 0.0613604 | 3.27E-10 | -0.0346 | 0.0648 | 0.593999 |
| rs34703115 | C | T | 34.58465476 | -0.616186 | 0.104778 | 4.08E-09 | 0.0688 | 0.078 | 0.378 |
| rs35000415 | T | C | 200.2294151 | 0.587787 | 0.041539 | 1.86E-45 | 0.0134 | 0.039 | 0.7307 |
| rs35251378 | A | G | 52.84420952 | -0.235722 | 0.0324266 | 3.61E-13 | -0.0378 | 0.0318 | 0.2343 |
| rs353608 | G | A | 44.22170731 | 0.18633 | 0.0280198 | 2.93E-11 | -0.0258 | 0.0281 | 0.3587 |
| rs3747093 | A | G | 57.8138428 | 0.262364 | 0.0345055 | 2.88E-14 | -0.045 | 0.0303 | 0.1377 |
| rs389884 | G | A | 460.9908042 | 0.928219 | 0.0432319 | 2.92E-102 | -0.0087 | 0.0537 | 0.8718 |
| rs4274624 | T | C | 293.2513845 | -0.559616 | 0.0326791 | 9.73E-66 | 0.0118 | 0.0332 | 0.723601 |
| rs4388254 | T | C | 39.25943866 | 0.378436 | 0.0603977 | 3.71E-10 | -0.0046 | 0.0441 | 0.9175 |
| rs4661543 | G | T | 41.94262961 | 0.274437 | 0.0423755 | 9.40E-11 | 0.0352 | 0.0569 | 0.5365 |
| rs4916215 | T | C | 43.15163271 | 0.223144 | 0.0339693 | 5.07E-11 | 0.0401 | 0.0362 | 0.268 |
| rs58688157 | G | A | 44.19823106 | -0.223144 | 0.0335647 | 2.97E-11 | -0.0437 | 0.0349 | 0.2103 |
| rs58721818 | T | C | 75.6557835 | 0.65752 | 0.0755941 | 3.38E-18 | -0.0093 | 0.1054 | 0.9294 |
| rs597808 | G | A | 30.40478329 | -0.162519 | 0.0294736 | 3.51E-08 | -0.0241 | 0.0284 | 0.3956 |
| rs6671847 | A | G | 47.13087409 | 0.198851 | 0.0289651 | 6.64E-12 | 0.0204 | 0.0282 | 0.4696 |
| rs6679677 | A | C | 52.39196829 | 0.336472 | 0.0464854 | 4.55E-13 | 0.0465 | 0.0393 | 0.2372 |
| rs6889239 | C | T | 76.51123259 | 0.277632 | 0.03174 | 2.19E-18 | 0.0054 | 0.0311 | 0.8633 |
| rs7097397 | A | G | 42.11576829 | -0.18633 | 0.0287118 | 8.60E-11 | -0.0359 | 0.0291 | 0.2171 |
| rs73050535 | T | C | 33.02355559 | -0.71335 | 0.124134 | 9.11E-09 | -0.0547 | 0.1986 | 0.782899 |
| rs73068668 | A | G | 29.96640854 | -0.314711 | 0.0574903 | 4.40E-08 | -0.0361 | 0.0506 | 0.4752 |
| rs7768653 | T | C | 48.6189269 | -0.207014 | 0.0296891 | 3.11E-12 | -0.0349 | 0.0282 | 0.2165 |
| rs7823055 | T | G | 150.1071876 | -0.350657 | 0.0286208 | 1.64E-34 | -0.0319 | 0.0282 | 0.2575 |
| rs7899626 | T | C | 30.06154268 | 0.182322 | 0.0332532 | 4.19E-08 | 0.0634 | 0.0288 | 0.0279698 |
| rs9852014 | G | A | 158.6275546 | 0.620577 | 0.0492727 | 2.26E-36 | -0.0266 | 0.0522 | 0.6101 |

**Supplementary Table 5**. Detailed information of instrumental variables used in the Mendelian randomization analysis of Psoriasis on TMD (significant level of p < 5e-08). Abbreviations: TMD, temporomandibular joint disorders.

| **SNP** | **effect_allele** | **other_allele** | **F-statistic** | **associated with Psoriasis** | | | **associated with TMD** | | |
| --- | --- | --- | --- | --- | --- | --- | --- | --- | --- |
|  |  |  |  | **beta** | **se** | **pval** | **beta** | **se** | **pval** |
| rs11085725 | T | C | 46.84896948 | -0.00166753 | 0.000243626 | 7.70016E-12 | -0.0384 | 0.0318 | 0.2271 |
| rs11135059 | A | G | 113.8258817 | -0.00251427 | 0.000235663 | 1.39991E-26 | -0.0097 | 0.0304 | 0.7489 |
| rs11581607 | A | G | 34.10764232 | -0.00258652 | 0.000442884 | 5.19996E-09 | -0.0464 | 0.0667 | 0.4865 |
| rs11795343 | C | T | 40.70860256 | -0.00144443 | 0.000226388 | 1.79999E-10 | -0.0132 | 0.0289 | 0.6483 |
| rs2111485 | G | A | 37.26953164 | 0.00138038 | 0.000226111 | 0.000000001 | 0.0118 | 0.0283 | 0.6761 |
| rs28362345 | C | T | 131.040418 | -0.00271983 | 0.000237596 | 2.39994E-30 | 0.022 | 0.0299 | 0.4625 |
| rs28732109 | A | G | 2227.0464 | 0.0155997 | 0.000330561 | 1E-200 | 0.0129 | 0.0474 | 0.7852 |
| rs28998802 | A | G | 33.64637007 | 0.00186972 | 0.000322335 | 6.59994E-09 | 0.0192 | 0.0361 | 0.5958 |
| rs33980500 | T | C | 44.67289573 | 0.00280706 | 0.000419981 | 2.29985E-11 | 0.0442 | 0.054 | 0.4139 |
| rs4112787 | T | C | 49.86327082 | 0.00164731 | 0.000233284 | 1.59993E-12 | -0.0002 | 0.0295 | 0.9953 |
| rs582757 | T | C | 31.03173247 | -0.00137827 | 0.000247418 | 2.5E-08 | -0.0326 | 0.0297 | 0.2721 |
| rs632376 | G | A | 34.49308093 | -0.00131604 | 0.00022408 | 4.30002E-09 | 0.0507 | 0.0282 | 0.07246 |
| rs7951925 | G | A | 31.12546875 | -0.00127836 | 0.000229137 | 2.39999E-08 | 0.0496 | 0.0283 | 0.07935 |
| rs8016947 | G | T | 52.64538821 | 0.00161674 | 0.000222823 | 4.00037E-13 | 0.0132 | 0.0282 | 0.6409 |
| rs842636 | A | G | 35.54447158 | -0.0013276 | 0.00022268 | 2.5E-09 | 0.0191 | 0.0286 | 0.5038 |
| rs848 | C | A | 39.81814355 | 0.00179998 | 0.000285251 | 2.80001E-10 | -0.0324 | 0.0291 | 0.2661 |
| rs892666 | C | T | 111.2207235 | 0.00258785 | 0.000245384 | 5.30029E-26 | -0.002 | 0.0311 | 0.949 |
| rs9277937 | C | T | 57.61686885 | 0.00285115 | 0.000375617 | 3.19963E-14 | -0.016 | 0.0597 | 0.7893 |

**Supplementary Table 6.** Detailed information of instrumental variables used in the Mendelian randomization analysis of TMD on five AIDs (significant level of p < 5e-06). Abbreviations: TMD, temporomandibular joint disorders; RA, rheumatoid arthritis; MS, multiple sclerosis; AS, ankylosing spondylitis; SLE, systemic lupus erythematosus; AIDs, autoimmune diseases.

| **SNP** | **effect_allele** | **other_allele** | ***F*-statistic** | **associated with TMD** | | | **associated with RA** | | | **associated with MS** | | | **associated with AS** | | | **associated with SLE** | | | **associated with** **Psoriasis** | | |
| --- | --- | --- | --- | --- | --- | --- | --- | --- | --- | --- | --- | --- | --- | --- | --- | --- | --- | --- | --- | --- | --- |
|  |  |  |  | **Beta** | **se** | **P-value** | **Beta** | **se** | **P-value** | **Beta** | **se** | **pval** | **Beta** | **se** | **P-value** | **Beta** | **se** | **P-value** | **Beta** | **se** | **P-value** |
| rs12802125 | T | C | 23.51755128 | -0.1901 | 0.0392 | 1.28E-06 | -0.0304592 | 0.0256438 | 0.31 | 0.000327384 | 0.1720786 | 0.9922235 | NA | NA | NA | -0.040822 | 0.042748 | 0.339605723 | -0.00011533 | 0.158638 | 0.699999936 |
| rs1403910 | A | C | 24.39355358 | -0.1457 | 0.0295 | 7.89E-07 | -0.0202027 | 0.0153842 | 0.3 | 0.05706132 | 0.6505327 | 0.02917889 | NA | NA | NA | -0.0295588 | 0.0347266 | 0.39466626 | 0.000110611 | 0.638003 | 0.630000654 |
| rs1555846 | T | C | 21.75013224 | 0.3148 | 0.0675 | 3.06E-06 | 0.0295588 | 0.0194387 | 0.29 | -0.009265639 | 0.05075793 | 0.869958 | NA | NA | NA | -0.040822 | 0.060467 | 0.499605258 | NA | NA | NA |
| rs72738853 | C | T | 22.24912974 | 0.6448 | 0.1367 | 2.40E-06 | -0.113329 | 0.159071 | 0.47 | NA | NA | NA | NA | NA | NA | NA | NA | NA | NA | NA | NA |
| rs75675110 | A | G | 20.87510115 | -0.2088 | 0.0457 | 4.86E-06 | -0.0100503 | 0.0251383 | 0.75 | 0.002577808 | 0.1030519 | 0.9504781 | NA | NA | NA | 0.00995033 | 0.0294973 | 0.73586814 | -0.000202892 | 0.100426 | 0.580000009 |
